# A Study of Depression and Quality of Life in Patients of Lichen Planus

**DOI:** 10.1155/2015/817481

**Published:** 2015-02-23

**Authors:** Neena S. Sawant, Nakul A. Vanjari, Uday Khopkar, Satish Adulkar

**Affiliations:** ^1^Department of Psychiatry, Seth G.S. Medical College & KEM Hospital, Acharya Donde Marg, Parel, Mumbai 400012, India; ^2^Department of Dermatology, Seth G.S. Medical College & KEM Hospital, Acharya Donde Marg, Parel, Mumbai 400012, India

## Abstract

The precise cause of lichen planus is unknown, but the disease seems to be immunologically mediated. It is a psychocutaneous disorder. Due to scarcity of Indian studies in this field, we decided to study in patients of lichen planus the prevalence of depression and quality of life with comparison of the same in both the genders. Patients diagnosed as having lichen planus by consultant dermatologist were enrolled after informed consent and ethics approval. 45 patients were screened, of which 35 who satisfied the criteria were taken up for the study. A semistructured proforma was designed to collect the necessary information with administration of dermatology life quality index and Beck's depression inventory. While 25% were depressed with females being more affected than males, quality of life was impaired in more than 90% patients. Impairment was maximum due to symptoms and illness feelings, disturbed daily activities, or work and time consumption in treatment. There was a strong association between depression and impairment in quality of life in both the genders. This study helps in early identification of psychological problems in lichen planus patients and in planning their future course of management, hence reducing the lack of productivity and improving the prognosis and quality of life.

## 1. Introduction

Lichens are basically primitive plants composed of symbiotic algae and fungi. The term planus is Latin and it means “flat.” The British physician Erasmus Wilson was first to provide the name lichen planus as he first described it in 1869 and reported 6% of its prevalence in the general population. The characteristic appearance of lichen planus in the form of white striae that develops atop the flat surfaced papules was described by Wickham in 1895. Lichen planus (LP) is predominantly a disease of the middle aged and elderly population [1]. The precise cause of lichen planus is still unknown and it is a psychocutaneous disorder which affects the skin, mucosa, hair follicles, and nails. It is also associated with psychoneuroendocrine and psychoimmunological comorbidities [2]. Various viral, psychogenic, genetic, enzymatic, and immunological factors are listed as possible causes [3–7].

Brig et al. suggested that there is diversionary symbiosis between skin and psyche [8].

Psychological stress could have a negative impact on healthy skin, exacerbating or precipitating dermatological disorders suggesting the presence of interface between psychiatry and dermatology [9]. Due to visibility of the dermatological disorder, not only it is associated with cosmetic disfigurement but also it results in a variety of psychopathological problems affecting the patient, his/her family, and society [10].

The relation between psychiatry and skin diseases is bidirectional: psychiatric comorbidity influences the development and course of dermatologic diseases via the effects of stress, depression, and anxiety [11]. On the other hand, cosmetically disfiguring dermatologic diseases may cause significant psychosocial distress for patients [12]. Comorbid mental illness plays a substantial role in course, severity, response to therapy, and therefore the psychosocial well-being of the dermatologic patients. But the comorbid mental illness and its consequences on patients' quality of life have been underappreciated. Therefore, understanding the prevalence of psychiatric comorbidity and its potential effects on patients' lives may lead to changes in management approaches and ultimately to improve the patients' outcome. Patients with lichen planus often experience stressful events before the onset of the disease [13]. Major life events especially illness or death of dear one could precede or exacerbate cutaneous lichen planus [14].

Several researchers have also reported higher prevalence of mixed anxiety depression, social phobia, panic symptoms, obsessive thoughts, and dysthymia in patients with lichen planus [1, 15]. This research was therefore undertaken to study the prevalence of depression, quality of life in patients of lichen planus, and association between depression and quality of life with gender differences in a general hospital.

## 2. Methodology

The study was initiated in the dermatology outpatient department of a tertiary general hospital after institutional ethics committee permission. All patients were diagnosed as having cutaneous and/or mucosal lichen planus by the consultant dermatologist after clinical evaluation and skin/mucosal biopsy with a confirmation of the same on histological report. All the patients were informed about the nature of study and its applications and informed consent was obtained from patients who were willing to participate in the study. Patients were initially screened and only those above 18 years of age were enrolled in the study. Data collection was done over a period of 3 months. Those having medical comorbidity like infections, other immunological disorders, or existing psychopathology with ongoing treatment were excluded from the study. 53 patients were screened of which 35 were enrolled in the study. Seven patients of the 35 who were enrolled had oral and/or genital lesions whereas 28 patients had lesions over nails, limbs, and/or trunk.

A proforma was designed to enquire into the sociodemographic details like age, sex, marital status, religion, education, occupation, address, and income; it also included details about lichen planus lesions like location and duration, triggering factors like stress, autoimmunological disorders, exposure to drugs like gold, antimalarials, beta blockers, ACE inhibiters, NSAID, sulfonylurea, and so forth and family history of lichen planus, any previous medical or psychiatry illness, and medication history. All patients were also assessed on the following scales.

### 2.1. Dermatology Life Quality Index (DLQI) [16]

DLQI is a widely used questionnaire aiming to measure the impact of skin disease on adult patients' quality of life. The DLQI consists of 10 items covering six basic topics: symptoms and feelings, daily activities, leisure, work or school, personal relationships, and treatment, each rated on a 4-point Likert rating. DLQI calculates by collecting the sum of the scores of the above questions. Higher scores are associated with greater impairment of quality of life. DLQI score is displayed in percentage ranging from a minimum of “0” to a maximum of “30.” DLQI translations in Hindi and Marathi are available on the Internet and are permitted by the authors for use for clinical purposes.

### 2.2. Beck's Depression Inventory (BDI-II) [17]

This scale was devised by Beck in 1996. It contains 21 sentence groups aimed at assessing the level of depression. Observed depression signs are evaluated objectively. The 21 signs of depression included in the scale are sensibility, pessimism, sense of failure, sense of guilt, self-dissatisfaction, self-accusation, desire to commit suicide, hysterical weeping, seizures, nervous breakdown, social retreat, indecisiveness, conflicting self-image, sleep disturbances, tiredness, loss of appetite, loss of weight, psychological complaints, and lack of sexual desire. All the questions were developed based on signs normally seen in depressed individuals. Each category receives a score of 0–3 points. If a subject scores 0–16 points there is no depression, 17–20 points indicate mild depression and 21–30 moderate depression, and >31 points reveal severe depression. Studies using the scale indicate that the BDI is an appropriate method for assessing the signs and levels of depression in a given subject.

BDI translation and validation in Hindi and Marathi were done by an expert committee comprising of a local language translator, a Professor of psychiatry, and a Professor of community medicine. However, these results were not published.

All analyses were done with SPSS statistical version 17 at 5% significance.

## 3. Results 

The demographic variables revealed the mean age to be 45.15 ± 12.72 and 43.26 ± 14.52 years for males and females, respectively. Around 57% of our patients were males. Majority of the sample were Hindus with around 83% being married. All were from lower and middle socioeconomic class. 32 (91.42%) patients did not have any triggering factors before the start of illness. Seven (20%) out of 35 patients had lesions only over oral and/or genital areas, while rest 28 (80%) patients had lesions over other skin areas like limbs, trunk, neck, nails, and so forth. None of the patients had family history of lichen planus. Almost 69% of our patients had history of the disease for more than 1 year (Table 1).

When all were assessed for prevalence of depression on BDI, then 9 out of 35 (25.71%) patients were depressed. Gender differences for depression revealed females (77.77%) to be more depressed than males which was statistically significant (Fisher's test *P* value, 0.0216) (Table 2(a)). When depression was specifically assessed in oral and/or genital lichen planus patients, 3 (43%) out of 7 were depressed. On assessing for severity of depression as per the BDI, majority of the patients of both the genders had moderate to extreme depression (Table 2(b)).

When the patients were assessed for the dermatological quality of life, almost 91% had impairment in their dermatological quality of life with majority (62.5%) having moderate to severe impairment (Table 3(a)).

On assessing the areas of impairment as per DLQI, majority of the patients complained of itching/soreness in the lesions (88.5%), embarrassment or increased self-consciousness about having a disease (80%), and difficulties for both cream and oral drug treatment like oral steroids, isotretinoin, dapsone or topical steroids, tacrolimus ointment, and so forth (77%). 45% had difficulty in shopping as they avoided going out due to embarrassment, while 40% had difficulties in other social and leisure activities. Up to 37% had a change in their clothing habits in the form of wearing full sleeve clothes to hide the lesions. Parameters like difficulty in sports, work, or study and interacting with partner, friends, relatives, or sexual relations were encountered less frequently as compared to rest of the parameters (Table 3(b)).

On further comparing both the genders on the various domains a significant difference was noted only on the work and school domain with women being more affected than the males (*U* = 78, *P* = 0.0156) as compared to the other domains where no significant differences were seen (Table 3(c)).

When assessment for association of depression with quality of life was carried out, females showed highly significant findings on all the domains of DLQI whereas males also had highly significant findings on most of the domains of DLQI except for school, work, and personal relationships (Tables 4(a) and 4(b)).

## 4. Discussion

Psychodermatology is an interesting area of interface between psychiatry and dermatology because of bidirectional interaction between skin and brain, which probably is due to ectodermal origin of brain and most of the skin. Lichen planus is a disease of unknown aetiology that affects the skin, mucosa, hair follicles, and nails. Our findings about the mean age of onset, marital status, and gender ratio for lichen planus patients are in keeping with those of previous researchers [18, 19]. Akay et al. [18] in their study found mean age of lichen planus patients to be 46.93 ± 13.7 years, with 60% of the patients being males and 77% being married.

Psychosocial stress can induce autoimmune or inflammatory skin disorders through neuroendocrine and neuroimmune dysregulations. Patients with lichen planus do experience stressful events before the onset of the disease; major life events especially illness or death of dear one could precede or exacerbate cutaneous lichen planus. Burkhart et al. [20], Andreasen [21], Shklar [22], and Ivanovski et al. [23] found that stressful events preceded development of oral lichen planus lesions. This finding was also confirmed by the authors who investigated psychological disturbances in patients with lichen planus of the skin [13, 14, 24]. But majority of our patients did not report any triggering or precipitating factors preceding the lesions. Only 3 out of 35 patients had triggering factors where one patient had exacerbation of lichen planus after the death of dear one and two had exposure to antimalarial drugs before onset of lichen planus.

As a part of bidirectional interaction, dermatologic disorder may result in psychiatric morbidities like depression, anxiety, social phobia, panic symptoms, and negative impact on quality of life [2, 15, 25]. Hampf et al. [26] found that only 48.2% of oral lichen planus patients were mentally sane, whereas 21.4% had minor, 5.4% moderate, and 25% severe mental disturbances. The same authors found that patients during mental stress had exacerbation of oral lichen planus lesions. We also found that 3 of the 7 patients who had oral and/or genital lesions had depression. The prevalence of depression in previous studies of lichen planus varied from 21% to 53% [18, 25] which is in keeping with our finding of 25%, with a female preponderance [27]. Researchers have found the severity of depression in lichen planus to range from moderate to severe depression, namely, 7% to 53% [18, 25], whereas we found a higher prevalence of nearly 90% in our patients having moderate to severe depression. The reason for the same could be that there was a female preponderance in our study. Generally, females experience more intense depressive features because of the more stress experienced and have a greater reactivity to it with a higher rate of body dissatisfaction and low self-esteem [28–30].

Lichen planus is a dermatological condition which affects the quality of life. A study by López-Jornet and Camacho-Alonso [31] had demonstrated almost 100% impairment in quality of life in patients with lichen planus, whereas we found 91% of our patients having impaired quality of life. Our findings on dermatology quality life index showed greater impairment in areas of symptoms and feelings and daily activities. This was understandable due to the presence of the lesions on the exposed body parts due to which patients expressed psychological discomfort like embarrassment or increased self-consciousness about having the disease and going out in public. On comparing for gender differences on the various domains of DLQI, no significant differences were found except that females showed statistically significant impairment in work and school domain as compared to males. This could be again probably due to a greater reactivity to stress with a higher rate of body dissatisfaction and low self-esteem which is often seen in females. Females also have a greater cosmetic awareness than males and hence tend to avoid social involvement like going to school, office work, and so forth due to feeling of looking unattractive or being stared at by others [28].

Many also experienced treatment difficulties because of requirement of frequent follow up and long treatment duration which caused disturbances in their daily routine (Tables 3(a) and 3(b)).

In both the genders, depression and the various domains of quality of life were significantly associated. This indicates that depressed patients have significantly impaired quality of life and vice versa. Researches have shown that the impact of a skin disorder on the quality of life is considered in many cases to be a stronger predictor of psychiatric morbidities like depression, anxiety, sleep disturbances, and adjustment disorder than physician's evaluation of clinical disease severity [32, 33].

## 5. Conclusions

This study helps in the early identification of psychological problems in patients with lichen planus and in planning their future course of management. Awareness among the dermatologists about the effects of psychopathology on the lichen planus needs to be addressed. Early referral to the psychiatrist would not only result in the improvement of the mood but also help to cope with the lichen planus. It can reduce the lack of productivity and help to improve the prognosis and quality of life associated with lichen planus. Further research is however required to study the impact on the lichen planus with the improvement in depression and quality of life.

## Limitations

1. Our study had a small sample size and hence separate association for oral and cutaneous lichen planus with psychopathology and quality of life was not statistically possible.
2. The small sample size would also affect the interpretation of the results.
3. Larger sample size with analysis of different types of lichen planus with respect to psychopathology and quality of life would be therefore needed to study the impact.

## Figures and Tables

**Table 1:** Sociodemographic variables.

Variables		Male (*n* = 20)	Female (*n* = 15)	Total (*n* = 35)
Age	Mean	45.150	43.267	44.208
	SD	12.721	14.528	13.624

Marital status	Married	18 (90%)	11 (73.33%)	29 (82.86%)
	Unmarried	2 (10%)	4 (26.67%)	6 (17.14%)

Religion	Hindu	19 (95%)	15 (100%)	34 (97.14%)
	Other	1 (5%)	0 (0%)	1 (2.86%)

Triggering factor	Present	0 (0%)	3 (20%)	3 (8.57%)
	Absent	20 (100%)	12 (80%)	32 (91.43%)

Duration of illness	<1 year	13 (65%)	11 (73.33%)	24 (68.57%)
	>1 year	7 (35%)	4 (26.67%)	11 (31.43%)

**(a):** 

Depression	Males (*n* = 20)	Females (*n* = 15)	Total	*P* value (Fisher's test)
Present	2 (10%)	7 (46.67%)	9 (25.71%)	0.0216^*^
Absent	18 (90%)	8 (53.33%)	26 (74.29%)	

^*^Refers to significant.

**(b):** 

Depression Severity	Males (*n* = 2)	Females (*n* = 7)	Total (*n* = 9)
Borderline depression	0 (0%)	1 (14.28%)	1 (11.12%)
Moderate depression	1 (50%)	3 (42.86%)	4 (44.44%)
Severe to extreme depression	1 (50%)	3 (42.86%)	4 (44.44%)

**(a):** 

Impairment in QoL as per total DLQI	Males *n* = 20 (%)	Females *n* = 15 (%)	Total (%)	*P* value (Fisher's test)
Present	19 (95%)	13 (86.67%)	32 (91.43%)	0.5646
Absent	1 (5%)	2 (13.33%)	3 (8.57%)	

**(b):** 

	Domains	Males (*n* = 20)		Females (*n* = 15)	Total (*n* = 35)
Impairment of DLQI domains	Feelings and symptoms	Present	19 (95%)	14 (93.33%)	33 (94.29%)
		Absent	1 (5%)	1 (6.67%)	2 (5.71%)
	Daily activities	Present	9 (45%)	9 (60%)	18 (51.43%)
		Absent	11 (55%)	6 (40%)	17 (48.57%)
	Leisure	Present	8 (40%)	7 (46.67%)	15 (42.86%)
		Absent	12 (60%)	8 (53.33%)	20 (57.14%)
	Work and school	Present	1 (5%)	8 (53.33%)	9 (25.71%)
		Absent	19 (95%)	7 (46.67%)	26 (74.29%)
	Personal relationships	Present	7 (35%)	5 (33.33%)	12 (34.29%)
		Absent	13 (65%)	10 (66.67%)	23 (65.71%)
	Treatment	Present	15 (75%)	12 (80%)	27 (77.14%)
		Absent	5 (25%)	3 (20%)	8 (22.86%)

**(c):** 

Domain	Male *n* = 20		Female *n* = 15		Mann-Whitney *U* score	*P* value
	Mean	SD	Mean	SD		
Feelings and symptoms	3.250	1.585	3.333	1.988	144.50	0.8673
Daily activities	1.750	2.359	1.800	2.178	138	0.6994
Leisure	0.7500	1.293	1.133	1.457	129.50	0.500
Work and school	0.1500	0.6708	1.467	1.506	78	0.0156^*^
Personal relationships	0.5500	0.8870	1.200	1.897	138	0.6961
Treatment	1.250	1.070	1.733	1.163	114	0.2345
Total score	**6.700**	**6.736**	**10.400**	**11.673**	**129.50**	**0.5046**

^*^Refers to significant.

**(a):** 

Variable		Mean	SD	Spearman *r*	*P* value
BDI total		6.05	10.05		

DLQI domains	Feelings and symptoms	3.250	1.585	0.7370	0.0002^*^
	Daily activities	1.750	2.359	0.4626	0.0400^*^
	Leisure	0.7500	1.293	0.7129	0.0004^*^
	Work and school	0.1500	0.6708	0.3823	0.0962
	Personal relationships	0.5500	0.8870	0.4048	0.0767
	Treatment	1.250	1.070	0.5686	0.0089^*^
	Total score	**6.700**	**6.736**	**0.7614**	0.0001^*^

^*^Refers to significant.

**(b):** 

Variable		Mean	SD	Spearman *r*	*P* value
BDI total		17.733	19.370		

DLQI domains	Feelings and symptoms	3.333	1.988	0.8731	0.0001^*^
	Daily activities	1.800	2.178	0.8349	0.0001^*^
	Leisure	1.133	1.457	0.8610	0.0001^*^
	Work and school	1.467	1.506	0.7226	0.0023^*^
	Personal relationships	1.200	1.897	0.8390	0.0001^*^
	Treatment	1.733	1.163	0.6527	0.0083^*^
	Total score	**10.400**	**11.673**	**0.8605**	0.0001^*^

^*^Refers to significant.
